# Internal Migration and Depression Among Junior High School Students in China: A Comparison Between Migrant and Left-Behind Children

**DOI:** 10.3389/fpsyg.2022.811617

**Published:** 2022-03-30

**Authors:** Xiaodong Zheng, Yue Zhang, Wenyu Jiang

**Affiliations:** School of Economics, Zhejiang Gongshang University, Hangzhou, China

**Keywords:** internal migration, depression, junior high school students, migrant children, left-behind children, China

## Abstract

Using data from the China Education Panel Survey (CEPS), which was a nationally representative sample of junior high school students, this study examined the association of internal migration with depression among migrant and left-behind children, while exploring the moderating effect of gender difference and the mediating effects of social relationships. The results showed that migrant children had a significantly lower level of depression than left-behind children. Further, the difference in mental health between migrant children and left-behind children was more prominent for boys than girls. The mechanism analyses indicated that compared to left-behind children, internal migration positively predicted parent–child relationships and peer relationships of migrant children, which in turn reduced their depressive symptoms. Although migrant children suffered from a higher level of teacher discrimination than their left-behind counterparts, it had no significant relationship with depression after controlling for children’s social relationships with parents and peers. Our findings suggested that migrating with parents was helpful to reduce children’s depressive symptoms in comparison with being left behind. Therefore, actions should be implemented to reduce the occurrence of involuntary parent–child separation and the prevalence of children’s depressive disorders due to institutional constraints. In addition, necessary treatments are needed to improve the psychological wellbeing of disadvantaged children, especially among left-behind children with mental illness.

## Introduction

Due to the growing scope, complexity, and diversity of population movement, migration is becoming one of the deciding global issues of the 21st century (International Organization of Migration, 2020). With the largest population in the world, China’s internal migration (including rural–urban and urban–urban migration) is a common phenomenon in the process of its urbanization and economic development (Cheng et al., 2014; Zhao et al., 2018). Due to the constraints of the household registration (*hukou*) system, which are often associated with local public resources and services, children of migrants are divided into two groups: migrant children and left-behind children. Migrant children refer to the migrant population under 18 years of age who leave their *hukou* registration place and move along with their parents to urban areas. Left-behind children are defined as children whose migrant parents, one or both of them, work in cities and leave them with a caregiver, such as grandparents, in their home communities (Zhou et al., 2015; Zhang et al., 2019a). According to the statistics released by the Ministry of Education of China, the number of migrant children amid their nine-year compulsory education period reached 14.06 million in 2017. Meanwhile, the number of left-behind children in rural areas was 15.51 million (Ministry of Education of China, 2017). Besides the geographical movement of residence, internal migration may also profoundly affect the psychological health of the children of migrants due to the change of living environments and lifestyles (Zhao and Yu, 2016; Zhang et al., 2019b). In terms of left-behind children, although the remittances from migrant parents could increase human capital investment in children’s education and health, they may still suffer from mental disorders because of a lack of parent–child interaction and supervision, as well as a higher risk of peer victimization (Wen and Lin, 2012; Yue et al., 2020; Wang and Zhang, 2021). In regard to migrant children, migrating with parents not only helps avoid psychological risks caused by parent–child separation, but also provides an opportunity to broaden horizons, make new friends, and utilize local social resources and services in urban areas. However, migrant children who have not obtained local *hukou* may also be emotionally vulnerable when they encounter social exclusion and discrimination induced by structural barriers (Cui and To, 2019; Fang, 2020; Jiang and Dong, 2020). As such, it is of significance to investigate the relationship between internal migration and children’s mental health problems to develop targeted strategies to improve the wellbeing of children.

Previous studies have investigated the associations of internal migration with the psychological wellbeing of migrant and left-behind children through comparisons between the children of concern and their local peers. Specifically, they examined the difference between migrant and urban native children or the disparity between left-behind and non-left-behind children in the *hukou* registration place. On the one hand, A large body of past research indicated that migrant children had lower levels of subjective happiness, life satisfaction, as well as more psychological and behavioral problems than local children in cities (Yuan et al., 2012; Hu et al., 2014; Gao et al., 2015; Fang et al., 2016). A recent meta-analysis involving more than 4,600 migrant children and 5,000 urban children showed that the mental health of migrant children was significantly worse than that of their urban native counterparts (Zhang et al., 2019b). On the other hand, a considerable amount of literature compared the development of left-behind children with non-left-behind children and reported that parental migration negatively affected children’s educational achievement, physical health, emotional and behavioral outcomes, and health-related quality of life (Jia et al., 2010; He et al., 2012; Zhou et al., 2015; Murphy et al., 2016; Lei et al., 2018; Jin et al., 2020). However, relatively few studies have examined the difference in mental health between migrant and left-behind children, which is crucial to understand which group of children is more disadvantaged and capture the whole picture of the psychological development of children during the process of internal migration.

The life history (LH) theory has been increasingly used as a heuristic framework to understand the psychological wellbeing in recent years and it can also be applied to explain the mental health of children of migrant workers in China (Schwartz et al., 2010; Lu and Chang, 2019). According to the life history approach, human behaviors are the results of coordinated tuning of physiological and psychological systems (Chang and Lu, 2018). People’s life history trade-off strategy imposed by genetic and environmental pressures may increase their risk of developing a specific mental disorder or cluster of disorders (Del Giudice et al., 2015). Resource and extrinsic risk are two overarching environmental constraints for children’s development (Chang and Lu, 2018). Compared to their local non-left-behind counterparts, left-behind children face sudden resource abundance from their migrant parents’ remittance but they also encounter parental absence as a threat to safety. Parental migration may also negatively predict parent–child relationships due to the lack of parental care and supervision (Guo et al., 2015). In terms of migrant children, they often have lower levels of financial and social resources than urban native children. In addition, they are more likely to experience social exclusion in the new environment in comparison with their urban peers (Hu et al., 2014; Fang et al., 2016). Hence, when comparing the psychological wellbeing of migrant children with left-behind ones, the extrinsic risk dimension of the environment could be the main reason to explain the difference in mental health between migrant and left-behind children.

Past research has found that social relationships, which can be viewed as indicators of the extrinsic risk dimension of childhood environment, are distinctive between migrant and left-behind children (Fang et al., 2016). Social relationships refer to the connections between people who have personally meaningful interactions, such as relationships between family members and friends (August and Rook, 2013). Family, peer, and school are the three primary contexts of children’s social networks (Murphy, 2008; Fernández-Zabala et al., 2016). First, family is the immediate context for children’s development, and emotional support from parents is critical for children’s psychological health (Arregle et al., 2007; Zheng et al., 2020). Compared to left-behind children who confront parent–child separation, the parent–child relationship would be better for migrant children as they migrate with parents and have more parental emotional support and care (Wang et al., 2017; Zhang et al., 2019a). Second, positive peer relationship also contributes to children’s mental health because supportive social interactions with peers are beneficial to develop self-confidence and self-esteem, which subsequently affect their psychological wellbeing (Wong et al., 2009; Jiang and Wang, 2020). Previous studies found that, compared to their local peers, both migrant and left-behind children reported a higher level of bullying and peer victimization (Ye et al., 2016; Yan et al., 2019; Zhang et al., 2019a). However, many studies suggested that left-behind children are more likely to be the victims of bullying victimization than left-behind children due to a lack of social support from family (Xu and Xie, 2015; Wang and Liu, 2020). Third, teachers play a special role in children’s wellbeing by bridging home and school cultures and developing personal skills. Positive teacher–student interactions are conducive to children’s mental health and subjective satisfaction, while discrimination from teachers potentially threatens students’ psychological wellbeing (Xu et al., 2018). In comparison with left-behind children, earlier studies demonstrated that migrant children are more prone to be discriminated against by local teachers due to their non-local resident status, which is positively associated with their psychological distress (Wang et al., 2018). Overall, although migrant children are more likely to encounter teacher discrimination, they should have higher levels of parent–child relationships and peer relationships. As a result, migrant children may have better psychological health and fewer depressive symptoms than their left-behind counterparts.

In addition, some relevant evidence supported the notion that there might be gender differences when comparing the mental health of migrant children with left-behind children. On the one hand, girls may suffer more detrimental effects from discrimination (Lucas-Molina et al., 2018). Meanwhile, the son preference rooted in traditional Chinese culture may also inhibit psychological development among girls due to child maltreatment and neglect (Wang et al., 2019). On the other hand, the coping responses to adverse events are different between males and females. Compare to their male counterparts, females tend to internalize their behavior and pay more attention to their negative emotions and thoughts, which subsequently lead to more depressive symptoms (Gomez-Baya et al., 2017; Zheng et al., 2019; Cao et al., 2020). At the same time, evidence has also shown that it is more difficult for females than males to recover from mental disorders (Eid et al., 2019). Therefore, if migrant children perform better in mental health than their left-behind peers, then gender difference possibly moderate the influence, and the positive effect might be more prominent for males than females.

Given that few studies have examined the association and its possible mechanisms between internal migration and depression of children from a comparison between migrant and left-behind children, the present study aims to address this research gap using data from China Education Panel Survey (CEPS). According to the aforementioned theoretical analysis, this study is designed to test the following hypotheses:

*H1*: Migrant children have lower levels of depression than left-behind children.

*H2*: The relationship between internal migration and depression among migrant and left-behind children differs according to gender difference.

*H3*: Parent–child relationship, peer relationship, and teacher discrimination mediate the difference in depression between migrant children and left-behind children.

## Materials and Methods

### Data

The data used in this study were drawn from the latest publicly released wave of the China Education Panel Survey (CEPS) in the academic year 2014–2015. The CEPS, administered by the National Survey Research center at Renmin University in China, is a nationally representative longitudinal survey for junior high school students to explore the socioeconomic determinants of students’ development. The survey applied a stratified and multistage sampling design with probability proportional to size (PPS). First, 28 counties or districts were selected as primary sampling units. Second, four schools were randomly chosen within each selected county. Third, students in two classes from 8th grade in the second wave of survey within each chosen school were randomly selected. The CEPS survey, permitted by the ethics committee of Renmin University of China, collected four levels of data by in-person interviews, including student, parents and household, class, and school modules. These modules include topics such as the migration status of children and their parents, children’s mental health status, and children’s relationships with parents, teachers, and peers. These survey topics are beneficial for our study to investigate the mental health disparity between migrant and left-behind children and explore potential underlying mechanisms. The CEPS survey contents were informed with the written consent of the children, parents, and teachers. More details about the sampling, questionnaires, and other issues can be retrieved from the CEPS website (Available at http://ceps.ruc.edu.cn/English/Home.htm).

In the academic year 2014–2015, a total of 9,449 junior high school students were interviewed. According to the purpose of our study, we restricted our sample to children of the internal migrants, including migrant children and left-behind children. Furthermore, observations with missing values on key variables (i.e., migration status and depression) and covariates were excluded. Finally, the sample size for data analysis of this study was 2,871, with 1,430 migrant children and 1,441 left-behind children. The descriptive statistics (see Table 1) showed that 52.87% of the sample was males (*n* = 1,518), 63.81% had a rural *hukou* (*n* = 1831), 30.09% were at boarding school (*n* = 864), 34.13% were the only child in their families (*n* = 980). The average age of the sample was 14.619 years.

**Table 1:** Demographic characteristics of the participants (*n* = 2,871).

Variables	Frequency (*N*)	Percent (*%*)
Gender		
Male	1,518	52.87
Female	1,353	47.13
Age		
Range:13–17	*M* = 14.619	SD = 0.748
*Hukou* (Household registration)		
Rural	1831	63.81
Urban	1,039	36.19
School boarding		
Yes	864	30.09
No	2006	69.91
Only child		
Yes	980	34.13
No	1891	65.87
Self-reported health		
Poor	207	7.21
Moderate	911	31.73
Good	1753	61.06
Family economic condition		
Poor	723	25.18
Moderate	1995	69.49
Rich	153	5.33
Parental education		
Elementary school or below	318	11.08
Junior high school	1,359	47.34
Technical/vocational/senior high school	765	26.65
College or above	429	14.94

*M, Mean; SD, Standard deviation*

### Measures

#### Internal Migration

Internal migration was measured through identifying migrant children and left-behind children in this study, using survey information regarding the places of *hukou* registration and residences of children and parents, as well as their living arrangements. On the one hand, if students and their parents lived together and their *hukou* registration places were not their residential location (e.g., other counties or provinces), then the respondents were regarded as migrant children. On the other hand, if students lived in the *hukou* registration places and their parents lived and worked in urban areas in other counties or provinces, then the respondents were considered as left-behind children. In the data analysis of the present study, migrant children were coded 1, and left-behind children were coded 0 as the reference group.

#### Depression

Depression was assessed using a short version of the Center for Epidemiologic Studies Depression Scale (CES-D), which had five items about students’ negative emotional feelings in the past week, including feeling “depressed,” “unhappy,” “anxious,” “life is meaningless,” and “sad.” These questions had five response options: never, seldom, sometimes, often, and always, which were scored 0 to 4, respectively. The total score of the five items ranged from 0 to 20, was calculated as the depression score of students, with higher values indicating more depressive symptoms and lower levels of mental health (Zheng et al., 2020). Past research reported that the 5-item CES-D scale had high reliability among Chinese junior high school students (Jiang and Dong, 2020). In the present study, the depression scale’s reliability coefficient (Cronbach’s alpha) was 0.884, indicating a high internal consistency of the scale.

#### Parent–Child Relationship

The parent–child relationship was measured by two student-reported questions regarding the closeness of the relationship between children and their parents (Ma et al., 2020): “How is the general relationship between you and your father” and “How is the general relationship between you and your mother.” Each response to the parent–child relationship question was a 3-point Likert scale including “not close,” “average/moderate,” and “close,” which were coded from 1 to 3. The average score of the two items was calculated to measure the parent–child relationship, with high values implying high levels of parent–child relationship. Cronbach’s alpha of this scale was 0.658 in this study.

#### Peer Relationship

Peer relationship was measured using three student-perceived questions about the relationship with their peers within the same school. The respondents were asked to express their agreement with the following three statements: “Most of my classmates are nice to me,” “My class is in a good atmosphere,” and “I feel close to people in this school.” Each response ranged from “1 = strongly disagree” to “4 = strongly agree,” and the average score of the three questions was calculated to measure the overall peer relationship of students, with higher scores indicating better peer relationships of children. Cronbach’s alpha of the peer relationship scale was 0.749 in the current study.

#### Teacher Discrimination

Teacher discrimination was assessed by two parent-reported questions, following a previous study that investigated the association between teacher discrimination and depression of children (Jiang and Dong, 2020). Specifically, parents were asked the following questions: “Are the school teachers prejudiced against non-local students” and “Are the school teachers prejudiced against the parents of non-local students.” Each response to the two questions ranged from “1 = not prejudiced at all” to “4 = very prejudiced.” The average score of the two answers was computed as an overall indicator of teacher discrimination, with a higher score indicating a higher level of teacher discrimination. Cronbach’s alpha of this scale was 0.838.

#### Covariates

Covariates included demographic and family characteristics of the students in the present study, including gender (1 = male, 0 = female), age, *hukou* (1 = rural, 0 = urban), school boarding (1 = yes, 0 = no), only child (1 = yes, 0 = no), self-reported health (1 = poor, 2 = moderate, 3 = good), family economic condition (1 = poor, 2 = moderate, 3 = good), highest parental education level (from 1 = elementary school or below to 4 = college or above). Among the covariates, information on parental education was reported by the parents of students, and others were self-reported questions for 8th-grade students.

### Statistical Analyses

This study first summarized descriptive statistics of student and family characteristics, including frequency and percentage for categorical variables, and mean and standard deviation for continuous variables. Then, group comparisons on the depression and social relationships between migrant children and left-behind children were performed by sample t-tests to examine any differences in means between the two groups of children. Next, accounting for confounding factors, we conducted multivariate linear regression analysis on the relationship between internal migration and depression for the full sample (Model 1). In addition, the interaction effect between gender and internal migration for depression was examined by adding the interaction term to Model 1 (Model 2). If the interaction effect was significant, we stratified the multivariate linear regression analysis by gender and obtained the relationship between internal migration and depression for males (Model 2) and females (Model 4). Finally, to test the mediation hypotheses, a stepwise regression-based approach was developed to examine the possible channels through which internal migration affected depression in children (Baron and Kenny, 1986; Jiang and Wang, 2020). In the first step, we investigated the associations of internal migration and children’s social relationships, including parent–child relationship (Model 5), peer relationship (Model 6), and teacher discrimination (Model 7). In the second step, the relationships between potential mediators and depression of children were examined by adding social relationship variables together to Model 1 (Model 8). In addition, to estimate the relative contributions of the potential mediators, we further employed the Karlson–Holm–Breen (KHB) method to implement mechanism analysis (Karlson and Holm, 2011). KHB is an unbiased decomposition method that can be applied to linear and non-linear probability models. Given that the depression score was a continuous outcomes variable, we directly decomposed the total effect of children’s migration into direct effect and indirect effect and calculate the contributions of each component of potential mediators (indirect effects) in linear regressions. All regression models were adjusted for covariates and cluster effects on the school level. Effect sizes of all models were reported as beta (*β*) coefficients with 95% confidence intervals (95% CIs). All analyses were performed using Stata, version 15.1 (StataCorp, College Station, Texas).

## Results

### Descriptive Analyses

Table 2 presents the descriptive statistics of the key variables used in this study and group comparisons by sample t-tests between migrant and left-behind children. Overall, the average depression score of the participants was 6.238 (SD = 4.431). Meanwhile, migrant children had significantly lower levels of depression compared to left-behind children (migrant: *M* = 5.876, SD = 4.394; left-behind: *M* = 6.597, SD = 4.441; *p* < 0.001). Additionally, migrant children were observed to be significantly different from left-behind children in some social relationships. To be specific, in contrast to left-behind children, migrant children had a significantly better parent–child relationship (migrant: *M* = 2.560, SD = 0.472; left-behind: *M* = 2.451, SD = 0.503; *p* < 0.001) and peer relationship (migrant: *M* = 3.123, SD = 0.704; left-behind: *M* = 3.001, SD = 0.714; *p* < 0.001). Meanwhile, migrant children were subjected to a relatively high level of teacher discrimination against non-local residents compared to their left-behind counterparts (migrant: *M* = 1.163, SD = 0.464; left-behind: *M* = 1.132, SD = 0.423), but it was not statistically significant at 5% level (*p* = 0.055).

**Table 2:** Descriptive statistics of key variables of migrant and left-behind children.

Variables	Range	All (*N* = 2,871)	Migrant children (*N* = 1,430, 49.81%)	Left-behind children (*N* = 1,441, 50.19%)	value of *p*
Depression, M ± SD	0–20	6.238 ± 4.431	5.876 ± 4.394	6.597 ± 4.441	<0.001
Parent–child relationship, M ± SD	1–3	2.505 ± 0.491	2.560 ± 0.472	2.451 ± 0.503	<0.001
Peer relationship, M ± SD	1–4	3.062 ± 0.712	3.123 ± 0.704	3.001 ± 0.714	<0.001
Teacher discrimination, M ± SD	1–4	1.143 ± 0.445	1.163 ± 0.464	1.132 ± 0.423	0.055

*M ± SD, Mean ± standard deviation*.

Table 3 shows Pearson’s correlations of the main variables used in this study. Consistent with the above group comparisons, internal migration was significantly and negatively correlated with children’s depression, parent–child relationship, peer relationship, while it was not significantly correlated with teacher discrimination. Meanwhile, children’s relationships with parents and peers were negatively correlated with depression, whereas teacher discrimination was adversely associated with depressive symptoms.

**Table 3:** Correlation coefficient matrix for the main variables.

Variable	1	2	3	4	5	6	7	8	9	10	11	12
1.Parent–child relationship	−0.217^***^	—										
2.Peer relationship	−0.221^***^	0.265^***^	—									
3.Teacher discrimination	0.072^***^	−0.058^***^	−0.118^***^	—								
4.Internal migration	−0.088^***^	0.111^***^	0.078^***^	0.032^*^	—							
5.Gender	−0.078^***^	0.018	−0.069^***^	0.064^***^	0.016	—						
6.Age	0.060^***^	−0.037^**^	−0.070^***^	0.045^**^	−0.026	0.116^***^	—					
7.*Hukou*	0.019	0.017	−0.050^***^	−0.002	0.040^**^	0.014	0.121^***^	—				
8.Boarding	0.050^***^	0.004	−0.050^***^	0.075^***^	−0.269^***^	−0.000	0.107^***^	0.219^***^	—			
9.Only child	−0.043^***^	0.019	0.069^***^	−0.045^***^	−0.040^**^	0.084^***^	−0.131^***^	−0.310^***^	−0.173^***^	—		
10.Self-reported health	−0.259^***^	0.228^***^	0.191^***^	−0.049^***^	0.107^***^	0.039^**^	−0.065^***^	−0.007	−0.045^***^	0.031^*^	—	
11.Family economic condition	−0.085^***^	0.069^***^	0.118^***^	−0.021	0.170^***^	−0.022	−0.108^***^	−0.172^***^	−0.173^***^	0.116^***^	0.121^***^	—
12.Parental education	−0.069^***^	0.039^**^	0.104^***^	−0.058^***^	0.038^**^	−0.058^***^	−0.212^***^	−0.284^***^	−0.175^***^	0.280^***^	0.035^**^	0.227^***^

* *p < 0.1;*

** *p < 0.05;*

*** *p < 0.01.*

### Internal Migration and Depression

Table 4 reports the association between internal migration and depression among migrant and left-behind junior high school students. After adjusting the covariates and cluster effects at the school level, the results in Model 1 show that migrant children had significantly lower levels of depression compared to left-behind children (*β* = −0.398, 95% CI = −0.739 to −0.057, *p* < 0.05). As for the covariates, being male (*p* < 0.001), having better personal health (*p* < 0.001), and family economic condition (*p* < 0.05) were associated with less depressive symptoms, whereas being older age was correlated with higher levels of depression (*p* < 0.05).

**Table 4:** Multivariate linear regression analysis of the association between internal migration and depression among Chinese junior high school students.

	Overall (*N* = 2,871)				Male (*N* = 1,518)		Female (*N* = 1,353)	
Variable	Model 1		Model 2		Model 3		Model 4	
	Beta (*β*)	(95% CI)	Beta (*β*)	(95% CI)	Beta (*β*)	(95% CI)	Beta (*β*)	(95% CI)
Internal migration (ref: left-behind children)	−0.398^**^	(−0.739, −0.057)	−0.061	(−0.510, 0.389)	−0.678^***^	(−1.170, −0.186)	−0.034	(−0.512, 0.445)
Gender (ref: female)	−0.571^***^	(−0.914, −0.228)	−0.260	(−0.706, 0.186)	—		—	
Age	0.209^**^	(0.006, 0.412)	0.202^*^	(−0.000, 0.405)	0.275^**^	(0.003, 0.546)	0.094	(−0.220, 0.407)
*Hukou* (ref: urban)	−0.172	(−0.525, 0.182)	−0.182	(−0.534, 0.171)	−0.057	(−0.525, 0.411)	−0.381	(−0.923, 0.161)
Boarding (ref: no)	0.043	(−0.372, 0.457)	0.052	(−0.362, 0.466)	0.129	(−0.410, 0.667)	−0.063	(−0.642, 0.517)
Only child (ref: no)	−0.288^*^	(−0.630, 0.054)	−0.280	(−0.622, 0.061)	−0.270	(−0.733, 0.194)	−0.329	(−0.879, 0.221)
Self-reported health (ref: poor)								
Moderate	−2.058^***^	(−2.811, −1.305)	−2.071^***^	(−2.824, −1.318)	−1.605^***^	(−2.583, −0.627)	−2.540^***^	(−3.610, −1.469)
Good	−3.694^***^	(−4.425, −2.963)	−3.711^***^	(−4.441, −2.981)	−3.367^***^	(−4.303, −2.430)	−4.061^***^	(−5.138, −2.985)
Family economic condition (ref: poor)								
Moderate	−0.231	(−0.628, 0.166)	−0.248	(−0.646, 0.151)	−0.240	(−0.755,0.276)	−0.289	(−0.885,0.307)
Good	−0.840^**^	(−1.591, −0.088)	−0.870^**^	(−1.623, −0.117)	−0.543	(−1.649, 0.563)	−1.290^**^	(−2.318, −0.261)
Parental education (ref: elementary school or below)								
Junior high school	−0.070	(−0.609, 0.469)	−0.080	(−0.622,0.461)	0.393	(−0.362, 1.149)	−0.707^*^	(−1.546, 0.131)
Technical/vocational/senior high school	−0.332	(−0.926, 0.263)	−0.338	(−0.934, 0.257)	0.050	(−0.753, 0.853)	−0.902^**^	(−1.763, −0.042)
College or above	−0.388	(−1.082, 0.306)	−0.407	(−1.097, 0.283)	−0.328	(−1.340, 0.683)	−0.649	(−1.685, 0.387)
Internal migration ^*^ gender			−0.631^**^	(−1.241, −0.216)				
Intercept	7.172^***^	(4.043, 10.301)	7.140^***^	(4.002, 10.278)	4.990^**^	(0.742, 9.238)	9.780^***^	(4.927, 14.634)
*R* ^2^	0.084	0.085	0.075	0.091				
*F*	17.88^***^	17.32^***^	10.59^***^	9.27^***^				

*CI, Confidence interval. Robust standard errors are clustering at class level*.

* *p < 0.1;*

** *p < 0.05;*

*** *p < 0.01*.

### Moderating Effect of Gender Difference

**Figure 1:**
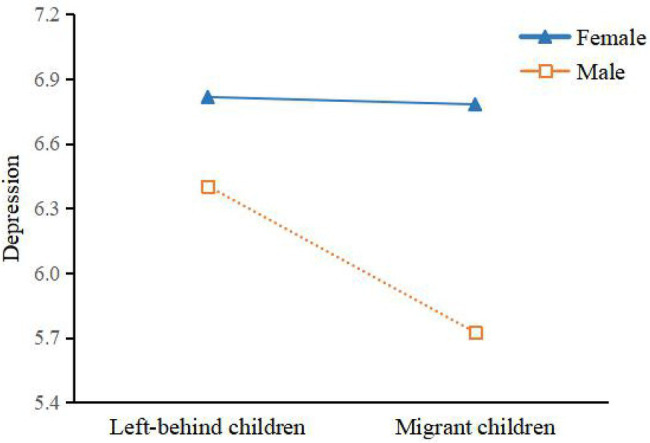
Gender difference as moderator of the relationship between internal migration and children’s depression.

The interactions between all covariates and internal migration for depression among migrant and left-behind children were also examined. The results (Model 2) demonstrated that only the interaction between gender and internal migration was significant (*β* = −0.631, 95% CI = −1.241 to −0.216, *p* < 0.05), indicating that internal migration has a significantly larger effect for boys to reduce symptoms of depression compared to girls. Simple slope analysis was performed for the descriptive purpose by plotting predicted depression of migrant and left-behind children, separately for boys and girls (Figure 1). The results demonstrated a more prominent difference in depression between left-behind children and migrant children for boys than that of girls. Stratified analysis by gender further confirmed the moderating effect of gender difference. As shown in regressions for male (Model 3) and female children (Model 4), internal migration was negatively and significantly associated with children’s depression for boys (*β* = −0.678, 95% CI = −1.170 to −0.186, *p* < 0.01), while it was not significant for girls (*β* = −0.034, 95% CI = −0.512 to 0.445, ns).

### Mediating Effects of Social Relationships

Table 5 shows the mediation analysis results, of which Model 5 to Model 7 demonstrate the first step estimates and Model 8 presents the second-step estimates. From the estimation results in the first step, internal migration was significantly associated with higher levels of parent–child relationship (*β* = 0.090, 95% CI = 0.044 to 0.136, *p* < 0.001) and peer relationship (*β* = 0.080, 95% CI = 0.005 to 0.155, *p* < 0.05). Meanwhile, internal migration also significantly predicted a higher level of teacher discrimination (*β* = 0.047, 95% CI = 0.006 to 0.088, *p* < 0.05). In terms of the second-step estimates, results showed that both parent–child relationship (*β* = −1.182, 95% CI = −1.531 to −0.833, *p* < 0.001) and peer relationship (*β* = −0.953, 95% CI = −1.234 to −0.672, *p* < 0.001) were negatively correlated with depression of children. In addition, teacher discrimination was positively associated with depression, but it was not statistically significant (*β* = 0.198, 95% CI = −0.220 to 0.617, ns). Besides, internal migration was no longer significant after controlling for potential mediators, indicating that parent–child relationship and peer relationship might be the main pathways to explain the association of internal migration with depression among migrant and left-behind children. Table 6 presents the estimated results of mechanism analysis using the KHB method. The results showed that parent–child relationship (33.49%, *p* < 0.01) and peer relationship (27.18%, *p* < 0.01) had significant mediating effects, explaining 60.67% of the total effect. Meanwhile, teacher discrimination was not a statistically significant component of indirect effects for the association between internal migration and depression among migrant and left-behind children. As such, among the potential mediators investigated in this study, parent–child relationship and peer relationship were the two leading channels underlying the difference in psychological health between migrant children and left-behind children.

**Table 5:** Results of mediation analysis for depression.

	Parent–child relationship		Peer relationship		Teacher discrimination		Depression	
Variable	Model 5		Model 6		Model 7		Model 8	
	Beta (*β*)	(95% CI)	Beta (*β*)	(95% CI)	Beta (*β*)	(95% CI)	Beta (*β*)	(95% CI)
Internal migration (ref: left-behind children)	0.090^***^	(0.044, 0.136)	0.080^**^	(0.005, 0.155)	0.047^**^	(0.006, 0.088)	−0.167	(−0.503, 0.170)
Gender (ref: female)	0.007	(−0.029, 0.044)	−0.107^***^	(−0.156, −0.057)	0.049^***^	(0.017, 0.082)	−0.672^***^	(−1.015, −0.330)
Age	−0.014	(−0.039, 0.012)	−0.022	(−0.060, 0.016)	0.006	(−0.015, 0.026)	0.193^*^	(−0.020, 0.406)
*Hukou* (ref: urban)	0.026	(−0.016, 0.068)	−0.003	(−0.067, 0.061)	−0.034^*^	(−0.072, 0.004)	−0.077	(−0.427, 0.273)
Boarding (ref: no)	0.046^*^	(−0.006, 0.098)	0.013	(−0.074, 0.100)	0.070^***^	(0.022, 0.118)	0.103	(−0.309, 0.515)
Only child (ref: no)	0.016	(−0.027, 0.060)	0.066^*^	(−0.006, 0.138)	−0.029	(−0.066, 0.007)	−0.173	(−0.523, 0.177)
Self-reported health (ref: poor)								
Moderate	0.156^***^	(0.072, 0.241)	0.172^***^	(0.049, 0.294)	−0.024	(−0.097, 0.049)	−1.882^***^	(−2.629, −1.134)
Good	0.327^***^	(0.243, 0.412)	0.395^***^	(0.275, 0.516)	−0.050	(−0.123, 0.024)	−3.055^***^	(−3.771, −2.339)
Family economic condition (ref: poor)								
Moderate	0.034	(−0.009, 0.077)	0.092^***^	(0.033, 0.151)	−0.010	(−0.049, 0.030)	−0.055	(−0.447, 0.338)
Good	0.018	(−0.066, 0.101)	0.178^***^	(0.067, 0.289)	0.032	(−0.058, 0.121)	−0.610	(−1.360, 0.140)
Parental education (ref: elementary school or below)								
Junior high school	0.016	(−0.046, 0.078)	0.110^**^	(0.025, 0.195)	−0.038	(−0.092, 0.016)	0.038	(−0.494, 0.570)
Technical/vocational/senior high school	0.027	(−0.041, 0.095)	0.146^***^	(0.055, 0.237)	−0.037	(−0.096, 0.022)	−0.134	(−0.726, 0.458)
College or above	0.067^*^	(−0.009, 0.143)	0.168^***^	(0.056, 0.279)	−0.062^*^	(−0.131, 0.007)	−0.138	(−0.828, 0.553)
Parent–child relationship							−1.182^***^	(−1.531, –0.833)
Peer relationship							−0.953^***^	(−1.234, –0.672)
Teacher discrimination							0.198	(−0.220, 0.617)
Intercept	2.325^***^	(1.931, 2.719)	2.891^***^	(2.320, 3.463)	1.089^***^	(0.773, 1.405)	12.129^***^	(8.706, 15.552)
*R* ^2^	0.063	0.066	0.018	0.132				
*F*	12.40^***^	12.64^***^	2.42^***^	21.72^***^				

*CI, Confidence interval. Robust standard errors are clustering at class level.*

* *p < 0.1;*

** *p < 0.05;*

*** *p < 0.01.*

**Table 6:** Mechanism analysis using the Karlson–Holm–Breen (KHB) method.

	Parent–child relationship	Peer relationship	Teacher discrimination
Estimated value (components of indirect effects)	−0.133^***^	−0.108^***^	0.010
	(−0.180, −0.071)	(−0.156, −0.045)	(−0.045, 0.029)
Mediating effects (%)	33.49%	27.18%	−2.63%
Total effect	−0.398 (−0.739, −0.057)^**^		
Direct effect	−0.167 (−0.503, 0.170)		
Indirect effect	−0.231 (−0.336, −0.126)^***^		

*95% confidence intervals in parentheses*.

** *p < 0.05;*

*** *p < 0.01*.

## Discussion

Using nationally representative data of junior high school students in China, this study investigated the association of internal migration with depression from the perspective of the comparison between migrant and left-behind children, while examining the moderating role of gender difference and the mediating role of social relationships. Consistent with our first hypothesis, this study found that internal migration was significantly and negatively associated with depression of children, indicating that migrant children had a lower depression level than their left-behind counterparts. Some previous studies suggested that migrating with parents had positive effects on children’s educational achievement, health performance, and behavioral outcomes in comparison with being left behind (Xu and Xie, 2015; Hu et al., 2018). Our study contributed to this literature by providing new evidence that internal migration was also positively associated with the psychological wellbeing among children of rural–urban and urban–urban migrants, though migrant children were still facing the potential challenge of social exclusion in the place of residence (Hu et al., 2014; Zhang et al., 2019b; Fang, 2020). This finding also supported the classical assimilation theory based on research regarding immigrants to the United States. The theory indicated that migration could help bridge the gap in wellbeing between migrants and their local-born peers, and migrants could achieve upward social mobility when they gradually adapted their lives and benefited from the opportunities and resources in the hosting environment (Greenman and Xie, 2008; Portes and Rivas, 2011).

Congruent with our second hypothesis, the present study confirmed that internal migration had a stronger association with depression for boys compared to girls. One possible reason is that females are more likely than males to have depressive disorders and present with internalizing symptoms due to biological sex differences. Such gender difference may not only result in a high prevalence of diagnostic depression among females, but also lead to an increased difficulty to reduce female depressive symptoms (Albert, 2015; Girgus and Yang, 2015; Gallo et al., 2018; Zheng et al., 2019). It is also possible that girls are at a higher risk of being exposed to violence and neglect than boys, particularly under the traditional Chinese culture of son preference (Wang et al., 2019; Zheng et al., 2019). As a result, in contrast to their left-behind peers, girls obtain fewer benefits than boys in mental health from internal migration because their parents may be inclined to invest more time, energy, and financial resources in sons rather than daughters (Lu and Tao, 2015; Kugler and Kumar, 2017).

In terms of the third hypothesis, the current study confirmed the mediating effects of parent–child relationship and peer relationship on the association between internal migration and depression among migrant and left-behind children. In other words, migrant children had better relationships with parents and peers in comparison with left-behind children, which in turn predicted lower levels of depression. This finding was consistent with previous studies indicating that left-behind children had fewer opportunities to derive emotional and social support from family, and they were at a higher risk to experience peer victimization than migrant children who lived with their parents (Jia and Tian, 2010; Zhang et al., 2019a; Wang and Liu, 2020). At the same time, social relationships were recognized as a crucial determinant of psychological health throughout one’s life course (Umberson et al., 2010; Jiang and Wang, 2020). In contrast, interestingly, our results suggested that teacher discrimination was not a significant mediator between internal migration and depression of children, which was contrary to the third hypothesis in this study. To be specific, although teacher discrimination was significantly associated with internal migration, it had no significant relationship with depression among children after controlling for the covariates and children’s social relationships with parents and peers. One possible explanation is that, compared to the relationship with teachers, children might be more concerned about the closeness with their parents and peers as social interactions involving family and friends played a central role in their social development and psychological wellbeing (Holder and Coleman, 2009; Fang et al., 2016). Previous studies also suggested that social support from family and friends was an essential protective factor for psychological distress caused by discrimination (Steers et al., 2019). Therefore, compared to teacher discrimination, parent–child relationship and peer relationship have more contribution to the mental health disparity between migrant children and left-behind children in our analyses.

This study contributes to the literature by revealing the difference in mental health status between migrant and left-behind children in China, as well as exploring the moderating effect of gender difference and mediating effects of social relationships. Although past research has indicated that, compared to their local peers living with parents, both migrant and left-behind children were considered as disadvantaged children in China (Hu et al., 2014). However, empirical studies regarding the mental health disparity between migrant children and left-behind children remain limited. The present study fills this research gap, which is helpful for the improvement of relevant policies to promote the development of underprivileged children and reduce social inequality in the long run. Our findings suggest that migrating with parents rather than being left behind is relatively beneficial for the psychological health of children of migrants. Although migrant children are more likely to be confronted by oppositional social environments and their assimilation process might be segmented (Xu and Xie, 2015), parent–child separation tends to be more detrimental for children’s psychological development as left-behind children reported more depressive symptoms than their migrant counterparts.

The findings of the present study have several implications for theory and practice. In terms of theoretical implications, the difference in wellbeing between migrant children and left-behind children should not be neglected when investigating the consequences of population migration. We encourage more research to examine the differences in other developmental outcomes (e.g., cognitive and non-cognitive skills) between the two groups of children. In addition, given the increasing number of children who have both migration and left-behind experience, such as return migration (Li et al., 2019), future research can also take these types of children into account to further investigate the developmental disparities among children with different migrant status and migration experience. In terms of policy implications, compared to migrating with parents, parent–child separation induced by institutional constraints are more detrimental for children’s psychological wellbeing. As such, policymakers should pay more effort to improve the household registration (*hukou*) system and population migration policy to reduce the prevalence of involuntary family separation (Zhou et al., 2015; Lei et al., 2018). In addition, evidence-based medical treatments and social supports are also needed for psychologically disadvantaged children, especially among left-behind children with mental disorders.

## Limitations

It warrants mentioning the limitations of this study. First, given the cross-sectional research design of the present study, the results should be interpreted as associations among variables rather than causal inference. Meanwhile, we may not fully measure and control for all potential confounders, such as specific information on children’s physical health and personality traits, in the analysis due to data constraints, which could compromise the validity of the results. Second, although showing a high level of reliability, the short version of the CES-D scale used in this study is a screening tool of depressive symptoms and it cannot provide a clinical depression diagnosis. Moreover, self-reported or parent-reported measures on the dependent variable and independent variables may also affect the validity of our findings due to potential measurement bias. Thus, our findings should be interpreted and generalized with caution. Third, since our sample was the only representative of the 8th-grade students with similar ages in China, the results may not be generalizable to the children of other ages and countries. Besides, although we used the latest publicly released CEPS data to conduct our study, the findings apply only to the time of the survey and they should be interpreted and generalized with caution. Therefore, we recommend more research to verify the results of this study with updated survey data. Future studies with more rigorous and comprehensive clinical diagnostic techniques and study design are also encouraged to further explore the association between internal migration and children’s psychological wellbeing.

## Conclusion

This study empirically investigated the association of internal migration with psychological health among Chinese junior high school students by comparing the depressive symptoms between migrant and left-behind children. We found that migrant children had a decreased level of depression in comparison with left-behind children. In addition, the relationship between internal migration and children’s depression was more significant for boys than girls. The mechanism analysis showed that parent–child relationship and peer relationship played mediating roles in the association between internal migration and children’s mental health. The findings of the present study underline the significance of reducing the occurrence of parent–child separation due to institutional barriers.

## Data Availability Statement

The raw data supporting the conclusions of this article will be made available by the authors, without undue reservation.

## Author Contributions

XZ: conceptualization. XZ and YZ: methodology and investigation. XZ, YZ, and WJ: validation, formal analyses, and writing—original draft preparation. All authors contributed to the article and approved the submitted version.

## Funding

This study was supported by the National Natural Science Foundation of China (Grant number: 72003173), Humanities and Social Science Fund of the Ministry of Education of China (Grant number: 20YJC790187), National Statistical Science Research Project (Grant number: 2021LY095), and Natural Science Foundation of Zhejiang Province, China (Grant number: LY21G030008).

## Conflict of Interest

The authors declare that the research was conducted in the absence of any commercial or financial relationships that could be construed as a potential conflict of interest.

## Publisher’s Note

All claims expressed in this article are solely those of the authors and do not necessarily represent those of their affiliated organizations, or those of the publisher, the editors and the reviewers. Any product that may be evaluated in this article, or claim that may be made by its manufacturer, is not guaranteed or endorsed by the publisher.
